# Performance and reproducibility on shuttle run test between obese and non-obese children: a cross-sectional study

**DOI:** 10.1186/s12887-017-0825-9

**Published:** 2017-03-09

**Authors:** Cristiane A. Moran, Maria Stella Peccin, Maria Teresa Bombig, Silvana Alves Pereira, Simone Dal Corso

**Affiliations:** 1 Federal University of São Paulo – UNIFESP, São Paulo, Brazil; 2 University of São Paulo – USP, Federal University of Rio Grande do Norte – UFRN, São Paulo, Brazil; 3 University Nove de Julho, São Paulo, Brazil; 4 Rua Dias de Toledo, 261 apto 1101 – Saúde, São Paulo, Brazil

**Keywords:** Child, Exercise, Exercise tolerance, Obesity, Physical fitness, Reproducibility of results

## Abstract

**Background:**

Due to the increasing prevalence of obesity among children, Shuttle Run Test (SRT) has been used as primary outcome for assessment of both physical performance and responses to different physical training programs. Thus, this study aimed to compare the performance on SRT between obese and non-obese children and the reproducibility of two SRTs carried out on different days.

**Methods:**

A cross-sectional study in which 40 children, aged from 8 to 10, were recruited from a public school. This study consisted of three visits in each school. On the first visit, we carried out a medical screening for recruited children. On the second visit, we applied the first SRT (SRT1), which was repeated on the third visit (SRT2, 24 hours apart).

**Results:**

There was a significant difference in the distance traveled by non-obese in comparison with obese children (mean difference: 88 meters and 95% of confidence interval: 21 meters to 156 meters). Time and distance traveled of 27 children were higher in the SRT1, whereas nine children traveled a greater distance and presented higher testing time on the SRT2, with only four children showing the same distance traveled in both tests. Although both groups presented with reduction from the SRT1 to SRT2, this reduction was not significant (non-obese: 342 ± 97 meters to 319 ± 106 meters, respectively; obese: 269 ± 91 meters to 246 ± 90 meters, respectively). In obese children, the distance traveled in the best SRT had correlation with weight (r = -0.495, *p* = 0.043) and BMI (r = - 0.602, *p* = 0.011). No correlation was observed in the non-obese children.

**Conclusions:**

Overweight children had lower performance in SRT. Although reproducible, the best performance was in the first test, which leads us to suggest applying only one test.

**Electronic supplementary material:**

The online version of this article (doi:10.1186/s12887-017-0825-9) contains supplementary material, which is available to authorized users.

## Background

Cardiorespiratory fitness can be objectively assessed by tests conducted in laboratory, but the need of expensive equipment limits its use in school environment. In this context, field tests might be an alternative for assessing cardiorespiratory fitness in school children, due to its low cost and its easy applicability, with the advantage that a big number of children can be assessed simultaneously [1, 2].

A recent systematic review reported that the shuttle run test (SRT) seems to be the most appropriate test to assess physical fitness in children and adolescents, with strong evidence that it presents good reproducibility [3]. Additionally, it has been demonstrated that SRT elicits maximal effort in children, which makes it a valid test to estimate the oxygen consumption in this population [4].

A new application for SRT is provided by a cohort study (10 years of follow-up) in which it was observed significant decline in cardiorespiratory fitness of English children about 7% for boys and 9% for girls. Decrease in SRT has been linked to body mass index (BMI) increase in children [5].

Due to the increasing prevalence of obesity among children, SRT has also been used as primary outcome for both assessment of physical performance and responses to different physical training programs. Considering BMI, obese children present worse SRT performance compared to non-obese at ages from12 to 18 years old [6]. However, studies with prepubescent obese children are scarce, and it is known that sexual maturation can influence the physical performance [7].

Previous studies have used PACER test for assessing of the performance in obese children [8, 9], but it does not seem to be suitable for this population as it is a very stressful test [8, 10], especially if we consider prepubescent obese children. In this context, few studies have focused on the prepubescent using the 20-metre SRT [11–13] which starts with a lower speed in comparison with PACER test, possibly contributing to optimize the performance of obese children [8].

The hypothesis to be tested is that the prepubescent obese children exhibit reduced performance compared to non-obese because of the difficulty of moving the body mass during a run test. Thus, this study aimed to compare the performance on SRT between obese and non-obese children and to test SRT reproducibility.

## Methods

### Study design and sample

In this cross-sectional study, a non-probability sample of 46 children, aged from 8 to 10 years old, were recruited from a public school of São Paulo, Brazil, with no history of cardiorespiratory diseases or musculoskeletal disorders. We excluded children with electrocardiographic changes at rest (*n* = 4), bone cancer (*n* = 1) and abdominal mass (*n* = 1), resulting a sample with 40 children. The study was approved by the Research Ethics Committee of Federal University of São Paulo (Protocol 1860/10) and those responsible for the child have signed an informed consent prior to the onset of assessments.

### Protocol

This study consisted of three visits in the school. On the first visit, we carried out medical screening for sample allocation. On the second visit, we applied the first SRT, which was repeated on the third visit, after a minimum of 24 hours without physical exertion.

### Evaluations

#### Assessment of socioeconomic status

Parents and/or those responsible for children answered a structured questionnaire on the purchasing power of the family and education level of the parents [14] (Additional file 1). The main criterion for Brazil's economic classification is the purchasing power of households. The score obtained from the questionnaire ranges from A to E, being the latter the lowest socioeconomic class.

*- Clinical evaluation*

Children underwent medical evaluation, composed of anamnesis, physical examination and electrocardiogram at rest in order to exclude cardiovascular diseases.

- Body mass index

Body mass index (BMI) was calculated by dividing body weight, expressed in pounds, by the square of child’s height in meters [15]. For body weight measurement, the child wore pants and t-shirt, without shoes. We used a digital scale (G-life®, Magna, China) with maximum capacity of 150 kg and 100 grams variation added to the extent of one decimal place.

Stature evaluation was carried out with a measure tape fixed on the wall, the children were barefoot and positioned with parallel feet, ankles close to each other, in upright position and arms along the body, with the head positioned so that the bottom of the eye socket was on the same plane as the ear external orifice. Subsequently, we calculated body mass index. Body mass index was classified according to gender and age, as eutrophic, underweight, overweight and obese children [15], according to World Health Organization. Subsequently the BMI was classified by percentile according to gender and age as follows: 15^th^ percentile - children with low weight, 50^th^ percentile - eutrophic children, 85^th^ percentile - overweight children and 97^th^ percentile - obese children [16].

For comparison of non-obese and obese children, sample was subdivided into two groups: underweight + eutrophic and overweight + obese, respectively.

- Shuttle run test

SRT was applied in multi-purpose sports courts, and schoolchildren performed the test individually, avoiding influence of competition among volunteers. Race speed was 8.0 km/h at the beginning of the test, with a 0.5 km/h increase every minute. Verbal encouragement was standardized every 1 minute by the same researcher with the follow phrases: "You're doing great." [17]. At the end of the test, we checked the number of excerpts conducted by the child and the number of complete snippets, which was converted to maximum speed of race according to each stage [18]. The test was stopped when the child was not able to reach the end of the path during the audio signal. Two SRT were performed (24h apart). The SRT1 was defined as the first test performed after the explanation of the evaluator about how it should be done, and SRT2 was performed to test the reproducibility. Both tests were done in the morning. The test with longer distance was considered to compare performance on the SRT between obese and non-obese children.

For heart rate (HR) measurement, we used the frequency meter Polar RS 800CX® model brand, the child was monitored at rest for one minute and during the entire SRT. HR at the end of the test was expressed in absolute values and percentage of predicted. HR was established in an expected value ≥ to 200 beats per minute (bpm) [19]. The following equation was used to express HR at the end of the SRT in percentage of maximum HR expected: HR, % predicted = (HR peak/200) x 100. Other than that, time and distance traveled in both SRT was recorded. To measure perception of effort, the modified Borg scale [20] was used immediately after the end of the tests.

#### Statistical analysis

Data were analyzed in a specific program for statistical analysis (SPSS-Statistical Package for the Social Sciences TM, version 13.0). First, Kolmogorov-Smirnov test was used to test data normality. All data presented with parametric distribution and were expressed as a mean and standard deviation. Unpaired Student's t-test was used for comparison of baseline characteristics between non-obese and obese children. Two-way repeated measures ANOVA with posthoc of Bonferroni test was used to compare the variables of SRT1 and SRT2 in non-obese e obese children. The better performance on SRT (test with longer distance) between non-obese e obese children was expressed in mean difference and limits of agreement. Also, the intraclass correlation coefficient was used to analyze the reproducibility of the two SRTs. A posteriori power analysis was calculated for the primary outcome (distance in SRT) by the G*Power software version 3.0.10. Spearman correlation was used to test the correlation between distance traveled and independent variables (age, weight, height, and BMI). A *p* < 0.05 was considered significant.

## Results

We studied 40 children, all classified as socioeconomic level E. Baseline characteristics are listed in Table 1.

**Table 1:** Baseline characteristics

Variables	Total sample (*n* = 40)	Non-obese (*n* = 23)	Obese (*n* = 17)
Sex (M/F)	15/25	5/18	10/7
Age (years)	8.7 ± 0.8	8.6 ± 0.7	8.9 ± 0.9
Weight (Kg)	36.3 ± 11.4	29.5 ± 4.0	45.4 ± 11.9
Height (m)	1.4 ± 0.7	1.4 ± 0.1	1.4 ± 0.1
BMI (Kg/m^2^)	18.6 ± 4.4	15.8 ± 1.5	22.2 ± 4.3

*Abbreviation*: *BMI* body mass index, *kg* kilogram, *m* meters, *kg/m* ^*2*^ kilogram per square meter

Regarding BMI, 20 children were classified as eutrophic (50th percentile), 3 undernourished children (15th percentile), 6 children as overweight (85th percentile) and 11 obese children (97th percentile).

As expected by groups allocation, obese group presented with significantly higher BMI than non-obese group (Table 1). Additionally, group 2 showed reduced distance and time values in SRT, with HR at peak test results similar to group 1. BMI may affect performance in STR, as seen in Table 2.

**Table 2:** Comparison of SRT1 and SRT 2 in non-obese e obese children

	Non-obese (*n* = 23)			Obese (*n* = 17)		
Variables	SRT 1	SRT 2	CCI (IC 95%)	SRT 1	SRT 2	CCI (IC 95%)
HR rest (bpm)	96 ± 21	94 ± 14	0.62 (0.09 – 0.84)*	104 ± 18	104 ± 13	0.68 (0.09 - 0.89)*
HRmaximum	195 ± 8**	194 ± 8**	0.02 (-1.44 – 0.591)	196 ± 13**	194 ± 11**	0.53 (-0.33 - 0.83)
Time (s)	142 ± 37	134 ± 40	0.70 (0.312 – 0.873)*	114 ± 36^†^	105 ± 36^††^	0.92 (0.76 - 0.97)*
Distance (m)	342 ± 97	319 ± 106	0.69 (0.287 – 0.868)*	269 ± 91^†^	246 ± 90^††^	0.92 (0.76 – 0.97)
Borg	6.4 ± 3.2	4.7 ± 3.2	0.62 (0.144 – 0.838)*	5.7 ± 3.2	6.0 ± 2.8	0.46 (-0.56 - 0.81)

* *p* < 0.05 for CCI SRT 1 vs. SRT 2 intragroup

** *p* < 0.05 vs. HR rest intragroup

†: *p* < 0.05 vs. SRT 1 non-obese

††: *p* < 0.05 vs. SRT 2 non-obese

Twenty seven children had longer distance in the SRT1, nine children in the SRT2, and only four children showed the same distance in both tests.

There was no difference in HR at rest and maximum HR for SRT 1 and SRT 2 in both non-obese and obese children (Table 2). Although both groups presented with reduction in the time and in the distance in the SRT 2, this reduction was not significant. No difference was observed for Borg scores in any conditions. Considering the best SRT, there was a significant difference in the distance traveled by non-obese in comparison with obese children (mean difference = 88 meters and 95% of confidence interval = 21 meters to 156 meters). The power of this analysis is 81.6% with an effect size of 0.367.

HR at the end of the test, expressed in percentage of prediction, matched 98 ± 5% on the SRT1 and 97 ± 5% on the SRT2, and 35% of the children reached predicted maximum heart rate ( ≥200 bpm). Maximum HR of 20 children was higher on the SRT1, four children remained the same on both tests and 16 children had lower values on the SRT2.

In obese children, the distance traveled in the best SRT had correlation with weight (r = -0.495, *p* = 0.043) and BMI (r = - 0.602, *p* = 0.011). No correlation was observed in the non-obese children.

## Discussion

Overweight and obese children presented the worse performance in the STR, represented by the shorter distance and time. Regarding reproducibility, this study showed that the best performance was made at the first shuttle run test.

These data reinforces the premise of worse performance in overweight population, which corroborates with the findings of another study conducted with ten year old children [17]. Performance at STR may also be characterized by achieved stage. Healthy children (12 ± 0.26 years old) reach, on average, 8.16 (±0.22) stages [21], however, eutrophic children classified as inapt (with performance inferior than expected) reach, on average, 2.8 (± 1.6) stages and obese children in the same conditions reach 2.6 (±1.7) stages [17].

In our study, overweight children showed lower performance which was similar to the observed in these studies, because children have reached 2.54 stages in STR. Characteristics related to low performance in physical tests with overweight children may be explained by a disproportional relationship between their body weight and their muscular composition [22], because the fat mass found in children with ages from 6 to 10 years old is 16.2% for the eutrophic ones and 32.7% on the obese ones [23].

In a study with obese adolescents (14 and 15 years old), the time of the SRT was on average 346 ± 156 seconds [24]. In our study with prepubertal children, the performance on SRT was even lower (130 ± 39 seconds), which leads us to infer that the sexual maturity influences the cardiopulmonary performance [25]. This is consistent with the findings that adolescents present better performance when compared with the pre-adolescents. Santos et al. showed that young girls (10 years) performed 22.2 ± 9.3 laps e boys 30.1 ± 15.0 laps in the SRT while older girls (14 years) performed 29.7 ± 12.5 laps and boys 48.9 ± 21.6 laps [7].

Test reproducibility is important for the possibility to meet physiological variability in this evaluation. Although there are various studies on Shuttle run test in children available [26–30], reports on reproducibility are scarce. Results found in this study confirm that there is no need to repeat SRT in healthy children because the better performance occurred in the first SRT. However, we cannot rule out that the reduced SRT2 performance may have occurred due to muscle fatigue, not only for peripheral muscle fatigue but also for central fatigue. A previous study has shown that obese girls (13.9 ± 0.9 years) presented early fatigue in comparison with their lean peers [30]. Although we have not observed difference between the performance from SRT1 to SRT2 intragroup, on average, obese children traveled shorter distance than non-obese children. This finding could be related to higher amount of type II fibers which are more prone to fatigue and the excessive work to move the body mass [30]. The latter can be supported in the present study by the negative and significant correlation between the traveled distance and weight and BMI only in the obese children, i.e. the higher the weight and BMI the lower the traveled distance in the SRT.

Cardiac stress during SRT may be represented by maximum HR to be reached. Children from this study showed 195 bpm, a value too similar to the one reported in Voss and Sandercock study, performed with children aged from 11 to 16 years old, that were either healthy or who did not present any alterations in the BMI, showing maximum effort with a 196 bpm HR [4]. Despite not measuring maximum oxygen consumption (maximumVO_2_), children have reached about 95% of their maximum heart rate and, as HR has a linear relationship with maximum oxygen consumption [31], it is possible to infer that the children have reached maximum metabolic stress.

STR is a valid and trustworthy measure [32], with the advantage of being used to evaluate a large number of participants simultaneously. Another advantage is the motivational aspect, because it involves running activity [33], which is considered to be an attractive activity among children. In clinical practice, field tests are more viable because they do not require high cost equipment and are simpler to be applied, because tests performed on a treadmill, for instance, depend on the child’s motor adaptation.

This study is innovative because it describes overweight children performance during a specific stress test and the need for its reproducibility, since a low cardiorespiratory performance is directly linked with obesity and changes in adipose tissue during childhood [34].

Sample selection by convenience is a limitation from this study; however, we were able to achieve similar results from previous studies. Even though the age group studied consisted only of children from 8 to 10 years old, this study contributes to the scientific collection, since there is a lack of studies investigating children with similar age. Besides, thin and fat mass composition was not taken into account, although the established protocol to measure body mass is commonly used in clinical practice, which has made possible for us to differentiate STR performance from eutrophic and overweight children. We suggest that future studies should be carried out by comparing performance in SRT with different socioeconomic levels.

## Conclusion

Overweight children have presented lower performance in shuttle run test. Even though it was reproducible, the best performance was during the first test, which leads us to suggest applying only one test.

## Additional file

Additional file 1: Questionnaire given to parentes. Socioeconomic questionnaire. Questionnaire on the purchasing power of the family and education level of the parents. (DOCX 13 kb)

## Abbreviations

BMI: body mass index
bpm: beats per minute
HR: heart rate
SRT: shuttle run test
VO_2_: oxygen consumption
