# Supplementary material for: Performance and reproducibility on shuttle run test between obese and non-obese children: a cross-sectional study
Source: BMC Pediatr. 2017 Mar 9;17:68. doi: 10.1186/s12887-017-0825-9 (PMC5345255; doi:10.1186/s12887-017-0825-9)
Supplement: Additional file 1: — Questionnaire given to parentes. Socioeconomic questionnaire. Questionnaire on the purchasing power of the family and education level of the parents. (DOCX 13 kb) [file 12887_2017_825_MOESM1_ESM.docx]

Socioeconomic questionnaire

The person in charge of the family:

( ) Did not study

() Incomplete primer

() Complete primer

() Incomplete Ginasial

() Full Gymnasium

() School incomplete

() Full school

( ) Incomplete higher

( ) Graduated

In your house you have:

DVD players? () No () Yes. How many? _____________

Washing machine? () No () Yes. How many? _____________

Refrigerator? () No () Yes. How many? _____________

Freezer ? () No () Yes. How many? _____________

Cars? () No () Yes. How many? _____________

Color TV? () No () Yes. How many? _____________

Bathrooms? () No () Yes. How many? _____________

Monthly employee? () No () Yes. How many? _____________

Radios? () No () Yes. How many? _____________
